# Cold Exposure After Exercise Impedes the Neuroprotective Effects of Exercise on Thermoregulation and UCP4 Expression in an MPTP-Induced Parkinsonian Mouse Model

**DOI:** 10.3389/fnins.2020.573509

**Published:** 2020-09-15

**Authors:** Yi-Ju Tsai, Yue-Cih Jhong, Shih-Hong Ching, Yu-Ching Liao, Cheng-Hsin Ching, Jih-Ing Chuang

**Affiliations:** ^1^Department of Physical Therapy, College of Medicine, National Cheng Kung University, Tainan, Taiwan; ^2^Department of Physiology, College of Medicine, National Cheng Kung University, Tainan, Taiwan; ^3^The Institute of Basic Medical Science, College of Medicine, National Cheng Kung University, Tainan, Taiwan

**Keywords:** treadmill exercise, cold exposure, brain temperature, uncoupling protein 4, Parkinson’s disease, MPTP

## Abstract

Moderate exercise and mild hypothermia have protective effects against brain injury and neurodegeneration. Running in a cold environment alters exercise-induced hyperthermia and outcomes; however, evaluations of post-exercise cold exposure related to exercise benefits for the brain are relatively rare. We investigated the effects of 4°C cold exposure after exercise on exercise-induced thermal responses and neuroprotection in an MPTP (1-methyl-4-phenyl-1,2,3,6-tetrahydropyridine)-induced Parkinsonian mouse model. Male C57BL/6J mice were pretreated with MPTP for five consecutive days and follow-up treadmill exercise for 4 weeks. After 1-h running at a 22°C temperature, the mice were exposed to a 4°C environment for 2 h. An MPTP injection induced a transient drop in body and brain temperatures, while mild brain hypothermia was found to last for 4 weeks after MPTP treatment. Preventing brain hypothermia by exercise or 4°C exposure was associated with an improvement in MPTP-induced striatal uncoupling protein 4 (UCP4) downregulation and nigrostriatal dopaminergic neurodegeneration. However, 4°C exposure after exercise abrogated the exercise-induced beneficial effects and thermal responses in MPTP-treated mice, including a low amplitude of exercise-induced brain hyperthermia and body temperature while at rest after exercise. Our findings elucidate that post-exercise thermoregulation and UCP4 expression are important in the neuroprotective effects of exercise against MPTP toxicity.

## Introduction

Moderate exercise promotes mitochondrial functions and upregulates survival factors that protect neurons from brain insults, including the movement disorders associated with Parkinson’s disease (PD) (Bayod et al., 2011; Wu et al., 2011; Koo et al., 2017). Our previous study demonstrated that chronic treadmill exercise prevents nigrostriatal dopaminergic neurodegeneration in an MPP^+^ (1-methyl-4-phenylpyridinium, a toxic metabolite of MPTP)-induced PD rat model by upregulating the nuclear-factor-E2-related factor 2 (Nrf2)-related antioxidant system (Tsou et al., 2015). We found that moderate treadmill exercise induces mild oxidative stress that leads to the activation of the endogenous Nrf2 antioxidant system to provide a defense against the lethal oxidative insults induced by MPP^+^. Similarly, induction of hyperthermia during exercise is unavoidable, and thus activation of thermoregulation after exercise to offset it is also essential. However, the role of thermoregulatory responses in exercise-induced neuroprotective functions is largely unclear.

During exercise, a more pronounced increase in brain temperature as compared with core body temperature was found (Kiyatkin, 2010; Kunstetter et al., 2014). Brain hyperthermia is attributed to excessive heat production due to increased brain metabolism and mitochondrial bioenergetics, as well as insufficient heat loss via blood circulation. Exercise training enhances ATP production via oxidative phosphorylation, mitochondrial mass by upregulating PGC-1α-regulated biogenesis, and heat production through uncoupling proteins (U) (Marques-Aleixo et al., 2015; de Oliveira Bristot et al., 2019). UCP1 can be found in brown adipose tissue for thermogenesis, while UCP2, UCP4, and UCP5 are expressed in the brain and involved in the brain’s neuroprotective functions (Kwon et al., 2010; Wu et al., 2014; de Oliveira Bristot et al., 2019). Voluntary exercise has been found to be able to elevate UCP2 expression in the hippocampus (Dietrich et al., 2008), while overexpressing UCP2 in the hypothalamus induces local brain hyperthermia (Conti et al., 2006), suggesting the involvement of UCP2 in exercise-induced brain hyperthermia. However, the effect of exercise on brain UCP4 and UCP5 expression remains uncharacterized.

The prevention of exercise-induced hyperthermia by running in a cold environment or the use of a pharmacological approach could potentially improve exercise capacity and endurance (Nybo, 2012; Fonseca et al., 2014), as well as increase the beneficial effects of exercise on cognitive performance and neurogenesis (Lee et al., 2014; Maynard et al., 2015). After the cessation of exercise, thermoregulatory responses, including an increase in heat loss via cutaneous vasodilation and sweating, need to be activated to offset the hyperthermia induced by exercise (Kenny and McGinn, 2017). Once the heat loss is disturbed, the restoration of core body temperature to resting levels can either be accelerated or delayed, and the impacts of exercise may also be changed (Friesen et al., 2014). On the other hand, cryotherapy after exercise has been shown to have promising therapeutic effects in terms of preventing high-intensity exercise-induced muscle inflammation and soreness (Lombardi et al., 2017). However, the effects of disrupting post-exercise thermoregulatory responses through mild cold exposure (a situation similar to when people like to stay in a cold environment after exercise) on moderate exercise-mediated neuroprotection remain unclear.

On the other hand, abnormal body and brain temperatures have been found in patients with neurodegenerative diseases. For example, the basal body temperature is low in transgenic Alzheimer’s disease (AD) mice (Vandal et al., 2016) and in acute MPTP-induced Parkinsonian mice (Freyaldenhoven et al., 1995; Jiao et al., 2015). Preventing hypothermia reduces neurodegeneration and improves brain functions in both of these neurodegenerative diseases (Jiao et al., 2015; Vandal et al., 2016). This is in contrast to the normal brain temperatures of AD patients (Sparacia et al., 2017), and a higher brain, but not body, temperature in individuals with PD (Sumida et al., 2015; Rango et al., 2016). Accordingly, long-term changes in body and brain temperatures during the nigrostriatal dopaminergic neurodegeneration that occurs in PD progression and the threats or benefits of hypothermia in PD patients and animal models require further examination.

In the present study, we used an MPTP-treated mouse model of PD to investigate the long-term changes in core body (peritoneal) and brain (striatal) temperature during the progression of MPTP-induced nigrostriatal dopaminergic neurodegeneration. We hypothesized that treadmill exercise would compensate the MPTP-induced thermal dysregulation and would be neuroprotective, while it was hypothesized that 4°C exposure after exercise would disrupt the thermal responses and neuroprotective effects of exercise. Because UCP4 expression may be involved in hypothermia-induced neuroprotection, and the effects of exercise on UCP4 expression remain unknown, we investigated whether brain UCP4 expression changes and contributes to the neuroprotective effects of exercise training and/or 4°C cold adaptation against MPTP-induced neurotoxicity.

## Materials and Methods

### Animals

All experimental protocols were in accordance with the National Institutes of Health Guidelines for Animal Research (Guide for the Care and Use of Laboratory Animals) and were approved by the National Cheng Kung University Institutional Animal Care and Use Committee (IACUC #105094). All efforts were made to ensure animal welfare. Male 4-week old C57BL/6J mice (body weight 18 ∼ 22 g) were used and were obtained from the animal facility of the Medical College, National Cheng Kung University, Tainan, Taiwan. The mice were housed in a 12/12-h light/dark cycle room at 22 ± 2°C and had free access to standard laboratory chow and water *ad libitum*.

### MPTP Treatment

**FIGURE 1:**
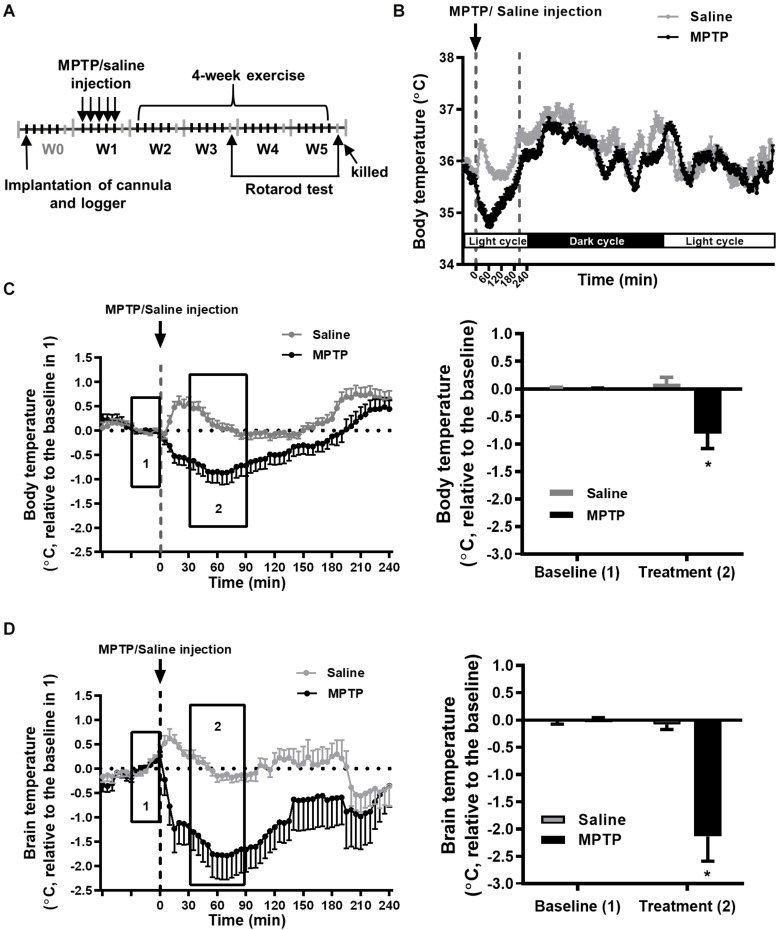
MPTP treatment induces a transient decrease in brain and body temperatures. **(A)** Time table of the logger implantation, MPTP injections, 4-week exercise period, and rotarod performance. The mice were intraperitoneally injected with MPTP (25 mg/kg/day) for five consecutive days in the first week (W1). The mean circadian core body (intraperitoneal) temperatures from 5 days of W1 for the MPTP- or saline-treated mice were real-time recorded by the loggers, as shown in **(B)**. The changes in core body **(C)** and brain (intrastriatal) temperature **(D)** from 1 h before to 4 h after MPTP or saline injection are shown in the left panels. Because the baselines (the average temperature 30 min before MPTP or saline injections, as shown in area 1) of absolute body and brain temperatures were not different among groups (Supplementary Table 1), the relative temperature changes shown in the *Y*-axis were calculated by deducting the respective baseline temperature for the statistical analysis. The average of the temperature changes in area 2 (30–90 min after the injection of MPTP or saline) is shown in the right panel of **(C,D)**. The values are shown as mean ± SEM and *n* = 12–14, with *p < 0.05, as compared with the saline-treated group using the unpaired *t*-test.

The mice were randomly divided into two groups: an MPTP-treated group and a vehicle solution-treated (0.9% sterile saline) group. We used an MPTP-induced mouse model of PD (Huang et al., 2017), where in the first week (W1, treadmill familiarization, Figure 1A) of the total experimental period of 5 weeks, the MPTP (25 mg/kg body weight, M0896, Sigma, St. Louis, MO, United States) mice were intraperitoneally injected once a day for five consecutive days. The vehicle control mice were injected with 0.1 ml of 0.9% saline. Two days after the last MPTP or saline injection, the mice were subjected to treadmill exercise or non-exercise for 4 weeks (second to 5th week; W2–W5, Figure 1A).

### The Protocols for Exercise Training and Post-exercise 4°C Cold Exposure

The rodent motor-driven treadmill (Model T408E, Diagnostic & Research Instruments Co., Taoyuan, Taiwan) used in this study was placed in a room with the temperature controlled at 22 ± 2°C. The 5-week exercise training program comprised 1 week of familiarization followed by 4 weeks of formal exercise training, as shown in Figure 1A and described previously (Wu et al., 2011). In the first week (W1) of the treadmill familiarization phase, 2 h after each MPTP injection, all mice were trained to run 10 min/day at a speed of 6–10 m/min for 5 days (6 and 8 m/min for 2 days, and 10 m/min for 1 day) to reduce the influences of handling and environmentally related stimuli. Electrical stimuli were not used to force the mice to run during the training program. After W1 treadmill familiarization, the mice were then randomly divided into exercise (Ex) and sedentary (Sed) control groups. In the second week (W2) of exercise training, the mice ran at a speed of 10 m/min for 20–60 min/day (an increment of 10 min/day), 5 days/week. In the following 3–5 weeks (W3–W5) of formal exercise training, the mice ran at a speed of 10 m/min (the running speed fulfilled the moderate exercise intensity criterion), 60 min/day, 5 days/week (Wu et al., 2011). The mice in the sedentary control group were placed on a stationary treadmill and did not run. After 1-h exercise or non-exercise from 13:00 to 14:00 during W3–W5, each group of mice were subdivided into two groups, one of which was immediately placed into a chamber for which the ambient temperature was set at 4°C for 2 h (1 mouse/cage) and the other of which remained in a room with the temperature control at 22°C for 2 h from 14:00 to 16:00. Subsequently, the mice were returned to their home cages (4–5 mice/cage) at 22°C room temperature. Accordingly, there were eight groups (*n* = 12–14, including three to four mice in each group for the measurement of body and brain temperatures) in this study: the saline-treated (vehicle solution of MPTP) sedentary group and post-exercise 22°C exposure (SS22) group, the saline-treated sedentary group and post-exercise 4°C exposure (SS4) group, the MPTP-treated sedentary group and post-exercise 22°C exposure (MS22) group, the MPTP-treated sedentary group and post-exercise 4°C exposure (MS4) group, the saline-treated exercise group and post-exercise 22°C exposure (SE22) group, the saline-treated exercise group and post-exercise 4°C exposure (SE4) group, the MPTP-treated exercise group and post-exercise 22°C exposure (ME22) group, and the MPTP-treated exercise group and post-exercise 4°C exposure (ME4) group.

### Measurement of Brain Temperature and Core Body Temperature

One week before the MPTP or saline injection, the mice were intraperitoneally anesthetized with sodium pentobarbital (60 mg/kg) and then mounted on a stereotaxic apparatus (David Kopf Instruments, Tujunga, CA, United States). For the measurement of brain temperature, a sterile stainless steel guide cannula (0.7 mm long, 1.1 mm OD, 19 gauge) was implanted into the right striatum (AP, 0.4 mm posterior to bregma; ML, 2 mm from the midline; and DV, 3 mm deep from the dura mater). The cannula was fixed on the skull with dental cement to allow the insertion of a thermocouple (T-type, 0.3-mm tip diameter) for the purpose of measuring the striatal temperature. The brain temperature data were collected with a hybrid recorder (Yokogawa HR1300, Japan) every 5 min during W1 of the MPTP injections (2 h before and 4 h after each MPTP injection) and during W3–W5 of exercise training (2 h before, 1-h exercise, and 3 h after exercise). Without recording, a sterile stainless steel obturator (0.8 mm OD, 21 gauge) was placed inside the cannula to keep the tract unblocked. The core body temperature of the mice was recorded using a miniature data logger (DSTnano; diameter 6 mm, length 17 mm, weight 1 g, Star-Oddi, Gardabaer, Iceland). The sterile logger was implanted into the abdominal cavity after the brain cannula implantation was completed. The loggers were programmed to record every 5 min within a 24-h period, and the data were downloaded at the end of the experiments. After 7 days of recovery, the MPTP injections were performed in W1, and follow-up exercise training was conducted from W2 to W5.

### Rotarod Test

The motor coordination of the mice was measured as the retention time on an accelerating rotarod apparatus (LE8200, Panlab S.L. Harvard Apparatus, Barcelona, Spain). Before the first training session, the mice were habituated on the apparatus at 4.0 rpm (revolution per minute) for 3 min and trained for three trials per day for three consecutive days before the MPTP injections. On the day of the test, the mice were placed on the rod with the speed accelerated from 4 to 40 rpm during a 5-min trial, where they received three test trials at 1-h intervals. The durations on the rod (mean latency to fall from the beam) were recorded and analyzed. The tests were performed 24 h after the second week of running exercise (W3) and 24 h after the last week of running exercise (W5).

### Histological Examination and Immunohistochemistry of the Brain

Twenty-four hours after the cessation of the 5-week exercise period, the mice were deeply anesthetized with urethane (1.8 g/kg, i.p.) and transcardially perfused with 0.9% saline. The brains of the animals were collected after perfusion and fixed in 4% paraformaldehyde for 48 h at 4°C and then dehydrated with a series of sucrose gradients (10–32%). Coronal brain sections containing the substantia nigra (SN; 20 μm) and striatum (ST, 30 μm) were made on a freezing Cryostat (CryoStar NX50, Thermo Scientific, MA, United States) and collected in cryoprotectant solution. The SN coronal brain sections were reacted with anti-tyrosine hydroxylase (TH; dilution 1:1,000, Millipore, Temecula, CA, United States) for DA neurons, and the ST brain sections were stained with anti-dopamine transporter (DAT; dilution 1:250, Santa Cruz, CA, United States) for the DA nerve terminals. The brain sections were incubated with appropriate secondary biotin-conjugated antibody and avidin–biotin–peroxidase antibody (ABC; Vector Laboratories, Burlingame, CA, United States) followed by the use of a glucose oxidase-diaminobenzidine method with or without nickel enhancement to detect antibodies.

### Cell Counting and Image Quantification

Cell counting was used to quantify the TH immunoreactive (TH^ir^) cells in the SN, as described previously (Wu et al., 2011; Tsou et al., 2015). In brief, the entire SN area was cut into an average of 36 coronal sections with a thickness of 20 μm. The number of TH^ir^ cells in the SN pars compacta (SNpc) was counted in every sixth section using a 100× objective under a microscope (Axioskop 2 plus, Zeiss, PA, United States). The number of TH-immunostained (TH^ir^) cells per section was determined by two other researchers who performed the cell counting blind to the treatment. The total number of TH^ir^ cells per SNpc was estimated from the total TH^ir^ cell number in six selected sections by dividing the ratio of selected and total sections (i.e., 6/36). For the quantification of DAT immunoreactivity, we defined the corpus striatum with a free hand tool in according to an optical fractionator. The average intensity of the DAT immunoreactivity in six striatal sections (every sixth section in the total of 48 sections) was measured using Image J (a free image analysis software), and each value was corrected for a non-specific background by dividing the optical density in the cortex.

### Immunoblotting

The UCP4 protein expression in the striatum was determined using immunoblotting, as described previously (Tsou et al., 2015). The striatum of the mice were collected 24 h after the cessation of 5 weeks of exercise and behavioral tests. Total protein extract was subjected to sodium dodecyl sulfate–polyacrylamide gel electrophoresis and transferred to a polyvinylidene difluoride membrane. After blocking, the membranes were incubated with anti-UCP4 antibody (Aviva Systems Biology, San Diego, CA, United States) and anti-β-actin antibody (Abcam, Cambridge, MA, United States) overnight at 4°C. Primary antibody binding was detected using horseradish peroxidase secondary antibodies and enhanced chemiluminescence reagents (Amersham Biosciences, Inc., United Kingdom). The optical intensity of the immunoblotted bands was quantified using a gel-scanning densitometer. The protein concentration was detected using a protein assay reagent (Bio-Rad Laboratories, Hercules, CA, United States) with bovine serum albumin as a standard.

### Statistical Analysis

The data were analyzed in GraphPad Prism 8.1 (GraphPad Software, San Diego, CA, United States) and were represented as means ± standard error of means (SEM). We used the unpaired Student’s *t*-test to compare the statistical significance between two independent groups. For multiple comparisons, we used a two-way ANOVA and the *post hoc* Tukey’s test. The eight groups were divided into two subgroups, the post-exercise 22°C- and 4°C-exposed groups, to run the two-way ANOVA independently, where the mice treated with saline or MPTP and the sedentary or exercised mice (four groups) were included in each subgroup. Statistical significance was set at *p* < 0.05.

## Results

### The Effect of MPTP Treatment on Core Body and Brain Temperatures

**FIGURE 2:**
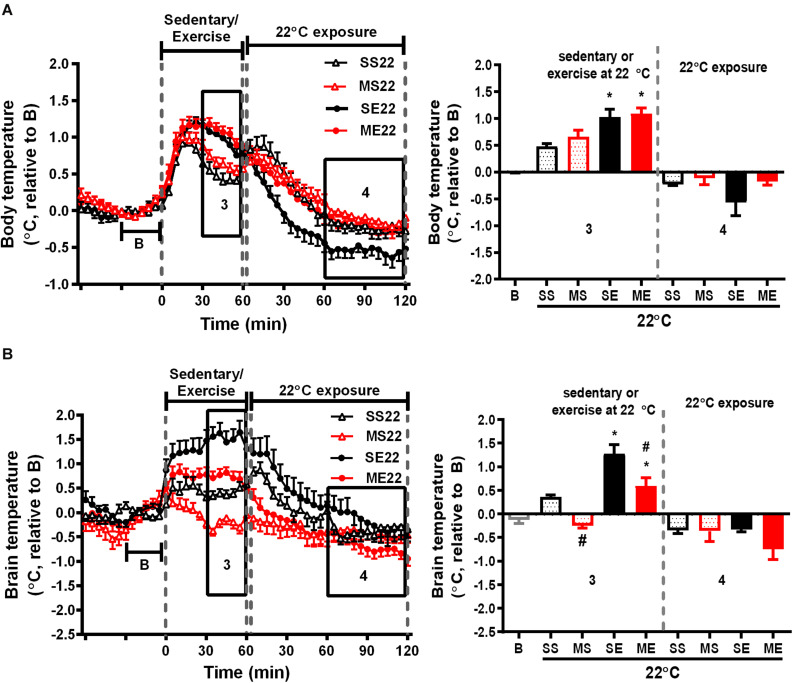
The effect of exercise on body and brain temperatures. The mean changes in body **(A)** and brain temperature **(B)** during the W3–W5 exercise training period were calculated from the daily temperature recordings, as shown in the left panels, from 1 h before to 1 and 2 h after exercise at a room temperature of 22°C. Because the baselines (the mean temperature in 30 min before running or not running, shown in **B**) of absolute body and brain temperatures were not different among groups (Supplementary Table 1), the relative temperature changes shown on the *Y*-axis were calculated by deducting the respective baseline temperature. The average of the temperature changes in area 3 (30–60 min after the onset of exercise) and area 4 (60–120 min after exercise with 22°C exposure) were calculated and are shown in the right panels. All groups are the mice remaining at a room temperature of 22°C (22) after running or not running. SS22 represents the saline-treated sedentary group; MS22 represents the MPTP-treated sedentary group; SE22 represents the saline-treated exercise group; and ME22 represents MPTP-treated exercise group. The values are shown as mean ± SEM and *n* = 3–4. Significance was found after the two-way ANOVA followed by the Tukey test, *p < 0.05, as compared with the corresponding sedentary group, and #*p* < 0.05, as compared with the corresponding saline-treated group.

**FIGURE 3:**
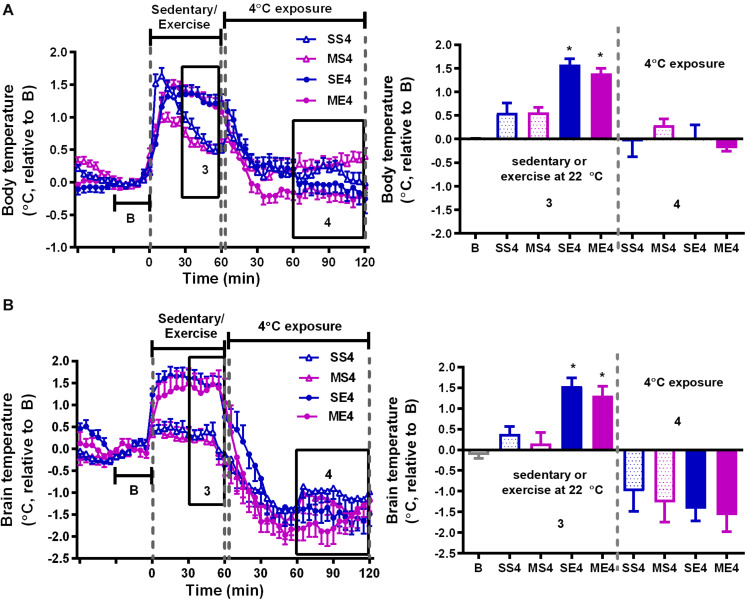
The effect of 4°C cold exposure after exercise on body and brain temperatures. Real-time changes in the body **(A)** and brain **(B)** temperatures were recorded daily from 1 h before to 1 h after exercise at a 22°C room temperature, followed by 2 h with 4°C exposure after exercise in the exercise training period from W3 to W5. The averages of the temperature changes in areas 3 and 4 were calculated as described in Figure 2 and are shown in the right panels. All groups (SS4, MS4, SE4, and ME4) are the groups exposed to a 4°C temperature (4) after 1-h running or not running at 22°C room temperature. SS is the saline-treated sedentary group; MS is the MPTP-treated sedentary group; SE is the saline-treated exercise group; and ME is the MPTP-treated exercise group. The values are shown as mean ± SEM, *n* = 3–4. Significance was found after the two-way ANOVA followed by the Tukey test, *p < 0.05, as compared with the corresponding sedentary group.

To better understand the changes in core body and brain temperatures during the progression of nigrostriatal dopaminergic neurodegeneration, we measured peritoneal and striatal temperature during the entire experimental period, as shown in Figure 1A, performed daily MPTP injections for a total of five injections in W1, and subjected the mice to daily 1-h exercise at 22°C and 2-h post-exercise 4°C/22°C exposure during W3–W5. We found that each MPTP injection induced an obvious decrease in core body (peritoneal) temperature within 60 min, which returned to the baseline at around 210 min, while only a transient, slight increase was found in the saline (MPTP vehicle)-treated mice due to the handling required for injection (Figure 1B). The average body temperature change was 35.25 ± 0.26°C (baseline, 30 min before daily saline or MPTP injection for 5 days in W1, as shown in box 1 of Figure 1C) to 35.19 ± 0.22 (mean temperature in the period of 30–90 min after saline injection in box 2 of Figure 1C) and 35.45 ± 0.13°C to 34.64 ± 0.41°C in the MPTP-treated mice. However, the hypothermia induced by the MPTP had a transient effect because the changes in the resting (circadian) body temperature were comparable in the saline- and MPTP-treated mice (Figure 1B). Because there were no between-group differences in the baselines of body (box 1 of Figure 1C) and brain temperatures (box 1 of Figure 1D) for the saline-treated (body temperature, 35.25 ± 0.26°C; brain temperature, 35.34 ± 0.22°C) and MPTP-treated (body temperature, 35.45 ± 0.13; brain temperature, 35.53 ± 0.14°C) groups, the body and brain temperature changes relative to the respective baselines were calculated and statistically analyzed. We found that the body and brain temperatures had significantly declined after MPTP treatment as compared with those in the saline-treated group (body temperature, −0.88 ± 0.28°C vs. 0.09 ± 0.11 in Figure 1C; brain temperature, −2.13 ± 0.45°C vs. −0.08 ± 0.09°C, Figure 1D, *p* < 0.05, using an unpaired *t*-test). The MPTP-induced brain hypothermia was more pronounced than the decreases in body temperature (−2.13 ± 0.45°C in brain temperature in Figure 1D vs. −0.88 ± 0.28°C in body temperature in Figure 1C, *p* < 0.05, using an unpaired *t*-test), although the detection systems for brain and body temperatures were different and were not calibrated with each other. Furthermore, the brain and body temperatures were measured daily during the formal exercise training period (W3–W5, 2–4 weeks after the MPTP treatment) to examine the long-term effects of MPTP on brain and body temperatures. We found no among-group differences in the baselines (the mean temperature in the 30 min before running or not running) in terms of absolute body and brain temperatures (Supplementary Table 1). The temperature changes relative to their respective baselines were calculated and statistically analyzed, as shown in Figures 2, 3. The results showed that the average changes in the daily brain temperature in the last 30 min of an hour at the stationary treadmill (to avoid the interference caused by moving to the stationary treadmill and exploratory activity) in the period 2–4 weeks after MPTP treatment were significantly lower than those of the mice pretreated with saline [Figure 2B, MS22 vs. SS22, *F*(1,9) = 27.47, *p* = 0.0005 using a two-way ANOVA and *post hoc* Tukey’s test], while no differences were found in the body temperatures for either group (Figure 2A). These results demonstrated that MPTP treatment not only induces a transient, high amplitude of decreases in core body and brain temperatures but also causes mild brain hypothermia found during the daily time spent on stationary treadmill and lasting for 4 weeks after the treatment.

### The Effect of Treadmill Exercise on MPTP-Induced Changes in Body and Brain Temperatures

We further clarified the effect of treadmill running on MPTP-induced thermal responses. In the saline-pretreated control mice, running at a room temperature of 22°C induced a significant increase in both body and brain temperatures compared with the non-running sedentary mice [body temperature changes of SE22 vs. SS22 as shown in Figure 2A, *F*(1,10) = 17.96, *p* = 0.0017 and brain temperature changes of SE22 vs. SS22 as shown in Figure 2B, *F*(1,9) = 50.94, *p* < 0.0001]. In the MPTP-pretreated group, the exercise-induced elevation in brain temperature, but not the increase in body temperature, was significantly attenuated compared with that in the saline-pretreated mice [Figure 2B, ME22 vs. SE22, *F*(1,9) = 27.47, *p* = 0.0005]. On the other hand, the low brain temperature in the MPTP-treated sedentary mice (MS22) while remaining at stationary treadmill was reversed in the mice who ran on the treadmill [Figure 2B, ME22 vs. MS22, *F*(1,9) = 50.94, *p* < 0.0001]. In addition, the average changes in the brain and body temperatures during the 2 h of post-exercise at 22°C were slightly declined below the baseline, but there were no differences among groups (Figure 2).

### The Effect of Post-exercise 4°C Exposure on Body and Brain Temperatures

We further examined the effect of recovery in 4°C environment after exercise on exercise-induced temperature changes in the mice treated with or without MPTP. In the 1-h treadmill running period at 22°C, the amplitude of exercise-induced body hyperthermia was significantly higher in the mice pretreated with saline after cold exposure (SE4) than it was in the mice without cold exposure (1.58 ± 0.12°C in SE4 in Figure 3A vs. 1.03 ± 0.15°C in SE22 in Figure 2A, *p* < 0.05 based on an unpaired *t-*test), while the increased brain temperatures in both groups were comparable (1.55 ± 0.20°C in SE4, as shown in Figure 3B vs. 1.27 ± 0.19°C in SE22, as shown in Figure 2B). However, the low brain temperature in the sedentary mice (MS22, Figure 2B) and reduced amplitude of exercise-induced brain hyperthermia (ME22, Figure 2B) were not found in the MPTP-treated mice with daily post-exercise exposure to a temperature of 4°C (MS4 and ME4 in Figure 3B). These results indicated that daily 4°C exposure enhanced exercise-induced body hyperthermia while preventing MPTP-induced thermal responses in the brain, including a low brain temperature during the time spent on the stationary treadmill and a reduction in exercise-induced brain hyperthermia. On the other hand, the brain, but not the body, temperatures during the 4°C cold exposure were significantly decreased in all mice with no differences among the groups (Figure 3B, no significance based on an unpaired *t*-test).

### The Effect of Treadmill Running and 4°C Exposure on Changes in Resting (Circadian) Body Temperature

**FIGURE 4:**
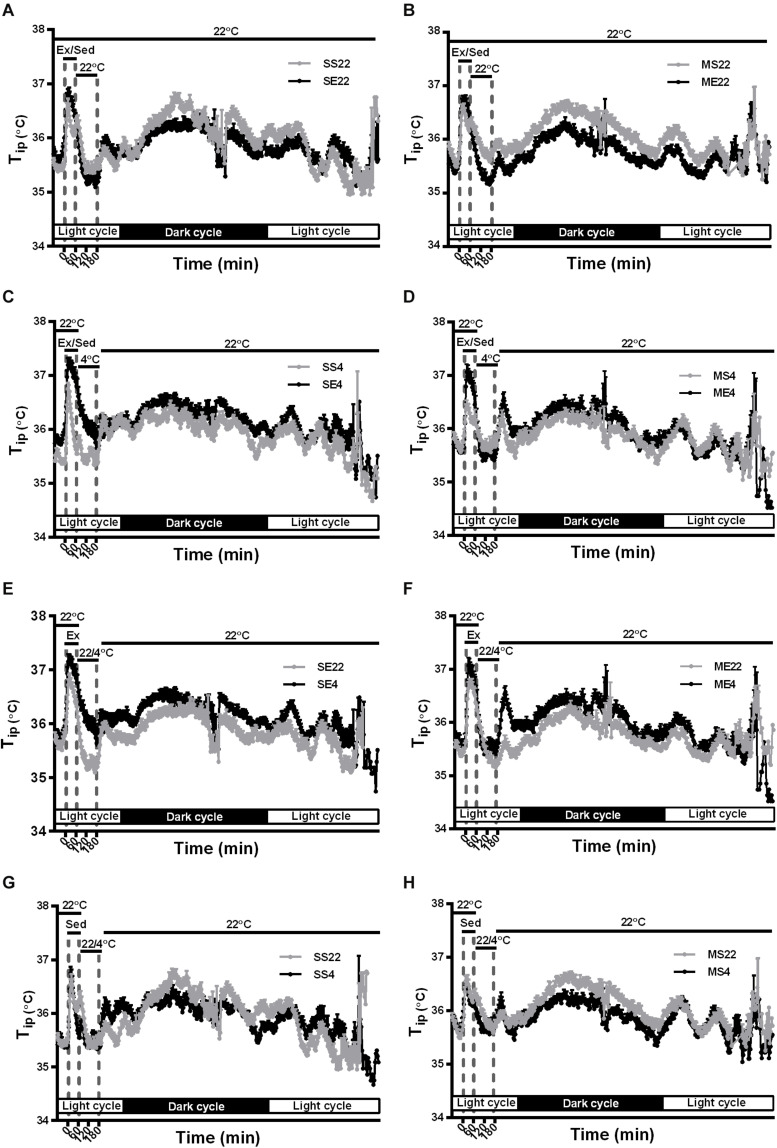
The effect of exercise and/or 4°C cold exposure on resting (circadian) body temperature. The average changes in the daily circadian peritoneal temperature (T_ip_) during the formal exercise training period from W3 to W5, where 5 days/week was calculated and shown in **(A–H)**. Panel **(A)** shows the circadian changes in the core body temperature (T_ip_) of the SS22 (saline-treated sedentary) and SE22 (saline-treated exercise) mice to present the between-group difference under the 22°C (22) exposure condition after exercise. Panel **(B)** shows the differences between the MS22 (MPTP-treated sedentary) and ME22 (MPTP-treated exercise) groups. Panel **(C)** shows the differences between the SS4 and SE4 groups with 4°C (4) exposure after exercise. Panel **(D)** shows the differences between the MS4 and ME4 groups. Panel **(E)** shows the differences between the SE22 and SE4 groups. Panel **(F)** shows the differences between the ME22 and ME4 groups. Panel **(G)** shows the differences between the SS22 and SS4 groups. Panel **(H)** shows the differences between the MS22 and MS4 groups. The values are shown as mean ± SEM and *n* = 3–4.

In addition to the temperature changes 1 h before, with 1 h of running or not running, and 2 h post-exercise 22/4°C exposure, as shown in Figures 2, 3, the average daily resting (circadian) body temperatures during the formal exercise training period of W3–W5, 5 days/week, were measured, as shown in Figure 4. We compared two groups at a time to understand the effect of a single factor (variant) on 24-h changes in body temperature. We examined the effect of exercise in the control mice saline-pretreated and exposed to 22°C and found that the overall resting body temperature in the mice with exercise training (SE22) was slightly lower than it was in the unexercised mice (sedentary mice, SS22, Figure 4A). In the MPTP-pretreated mice, the effect of exercise on the low body temperature (ME22 vs. MS22, Figure 4B) was more pronounced than it was in the saline-pretreated mice (SE22 vs. SS22, Figure 4A). However, this phenomenon of exercise-induced low resting body temperature disappeared when the mice were exposed to the 4°C chamber after daily exercise in the case of both the saline-pretreated (Figure 4C, SS4 vs. SE4) and MPTP-pretreated groups (Figure 4D, MS4 vs. ME4). We also examined the effect of post-exercise cold exposure on changes in exercise-induced body temperature and found that the resting body temperature in the post-exercise groups exposed to 4°C temperatures was higher than it was in the groups without cold exposure (Figure 4E, SE22 vs. SE4 with saline pretreatment and Figure 4F, ME22 vs. ME4 with MPTP pretreatment). The effect of daily cold exposure only on the resting body temperature was examined, and the results showed that there were no noticeable changes in the saline-treated sedentary mice with or without cold exposure (SS22 vs. SS4, Figure 4G). However, in the MPTP-treated sedentary mice, the resting body temperature was slightly decreased in mice with daily 4°C exposure compared with those kept at a temperature of 22°C (MS4 vs. MS22, Figure 4H). The results indicated that exercise induced a low resting body temperature, particularly in the MPTP-pretreated mice, which was disrupted by 4°C cold exposure after exercise.

### Cold Exposure After Exercise Impedes Exercise- or Cold-Induced UCP4 Upregulation and Prevention of UCP4 Downregulation After MPTP Treatment

**FIGURE 5:**
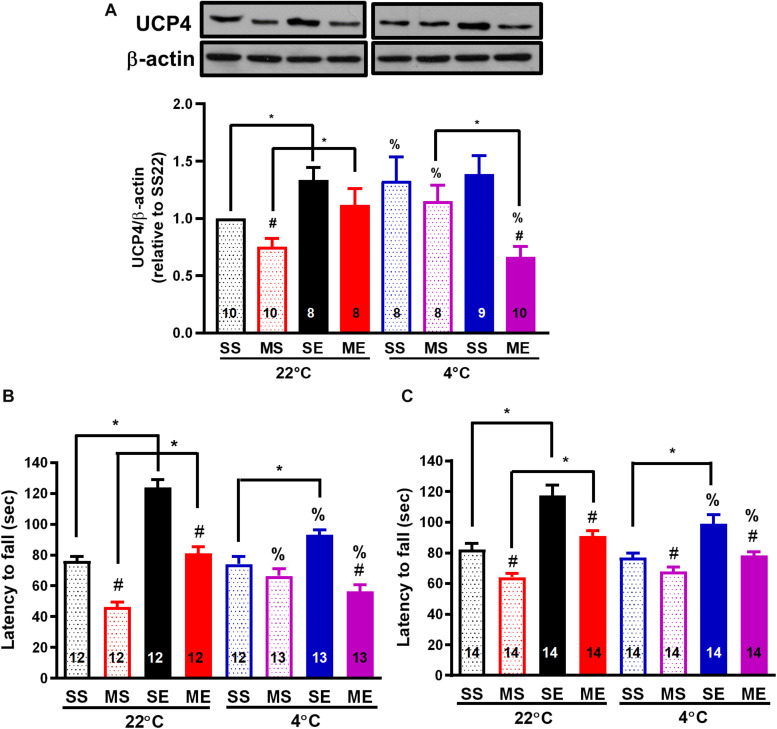
Post-exercise cold exposure impedes exercise-induced UCP4 upregulation and improvements in motor impairment after MPTP treatment. The striatum was collected for the determination of UCP4 protein expression using a western blot 24 h after the cessation of the fourth week of treadmill exercise. The representative western bands of UCP and β-actin are shown in the upper panel of **(A)**, and the optimal band intensity determined by densitometry is shown in the lower panel of **(A)**. A rotarod test was performed 24 h after the last run in the second week (**B**, W3) and fourth week (**C**, W5) of treadmill exercise to evaluate the motor balance and coordination of the mice. The mice were divided into eight groups, as described in Figures 2–4. The values are mean ± SEM, and the number in each column is the sample size (*n*). A comparison of the two groups indicated significance (*p < 0.05) and ^#^*p* < 0.05 indicated significance as compared with the corresponding saline-treated group using a two-way ANOVA and a *post hoc* Tukey’s test. The two-way ANOVA was performed separately for the data obtained from the post-exercise 22°C exposure groups (SS22, MS22, SE22, and ME22) and the post-exercise 4°C exposure groups (SS4, MS4, SE4, and ME4) as described in previous figures. A comparison with the corresponding 22°C exposure group indicated significance (^%^*p* < 0.05) after the unpaired *t*-test.

Because an increase in brain UCP4 and UCP5 mRNA expression after exposure to a temperature of 4°C had been demonstrated previously (Yu et al., 2000), and the effect of exercise on UCP4 and UCP5 expression remained uncharacterized, we investigated the protein expression of UCP4 and UCP5 in the striatum after the treatments. Exercise training for 4 weeks significantly increased UCP4 protein expression in the striatum compared with the saline-treated sedentary conditions [SE22 vs. SS22, *F*(1,34) = 16.97, *p* = 0.0002, Figure 5A]. In addition, UCP4 protein expression was also significantly (*p* < 0.05 based on an unpaired *t*-test) upregulated in the mice treated in the 4°C cold environment compared with mice under the 22°C condition (SS4 vs. SS22, Figure 5A). UCP4 expression was significantly downregulated in the MPTP-pretreated sedentary mice [MS22 vs. SS22, *F*(1,34) = 7.507, *p* = 0.0097]. The MPTP-induced UCP4 downregulation was blocked by exercise training or daily 4°C exposure (ME22 vs. SE22 and SE4 vs. SE22, Figure 5A). However, the exercise- or cold-induced prevention of MPTP-induced UCP4 downregulation disappeared in the MPTP-treated mice with post-exercise cold exposure (ME4 vs. ME22 and ME4 vs. MS4, *p* < 0.05, based on an unpaired *t*-test, Figure 5A). On the other hand, we found that the UCP5 expression was also decreased after MPTP treatment in 22 or 4°C exposed mice, but the effects of exercise on UCP5 expression were not as significant as the UCP4 expression (Supplementary Figure 1). These results indicated that exercise training or 4°C exposure induced striatal UCP4 upregulation and prevented MPTP-induced UCP4 downregulation, where the prevention was blocked by 4°C exposure after exercise.

### Exercise-Induced Motor Improvement and Neuroprotection Is Attenuated by Cold Exposure After Exercise

To evaluate the effect of exercise and/or cold exposure on MPTP-induced motor deficits, we conducted a rotarod test 24 h after the last run in the second (W3) and fourth week (W5) of exercise. The results showed that the riding time (latency to fall from the rotarod) was significantly reduced in the MPTP-treated sedentary mice compared with that in the saline-treated mice [MS22 vs. SS22, *F*(1,39) = 104.1, *p* < 0.0001 in Figure 5B and *F*(1,57) = 28.26 in Figure 5C, *p* < 0.0001], indicating that motor coordination was impaired from 2 to 4 weeks after MPTP treatment. In the mice undergoing exercise training, a significant increase in riding time was found compared with the sedentary mice (SE22 vs. SS22, *F*(1,39) = 80.30, *p* < 0.0001 in Figure 5B and *F*(1,57) = 55.39 in Figure 5C, *p* < 0.0001), and the MPTP-induced short riding time was prevented (ME22 vs. MS22) in both 2 (W3, Figure 5B) and 4 weeks (W5, Figure 5C) after exercise. In the mice with daily 4°C exposure, the prevention of an MPTP-induced decrease in riding time was only found at 2 weeks after cold exposure (MS4 vs. MS22, Figure 5B). On the other hand, in the mice undergoing 4°C post-exercise exposure, exercise-induced improvement in rotarod performance was impeded, as shown by the significant (*p* < 0.05 using an unpaired *t*-test) reduction in the exercise-induced riding time in the saline-treated (SE4 vs. SE22) and MPTP-treated (ME4 vs. ME22) mice (Figures 5B,C). Meanwhile, we also performed an open-field test before the rotarod test and found that the total distance traveled in 5 min was not different among the treatments (data not shown). These results indicated that treadmill exercise training or daily cold adaptation can improve MPTP-induced motor impairment, while post-exercise cold exposure suppresses the beneficial effects of exercise on motor function.

**FIGURE 6:**
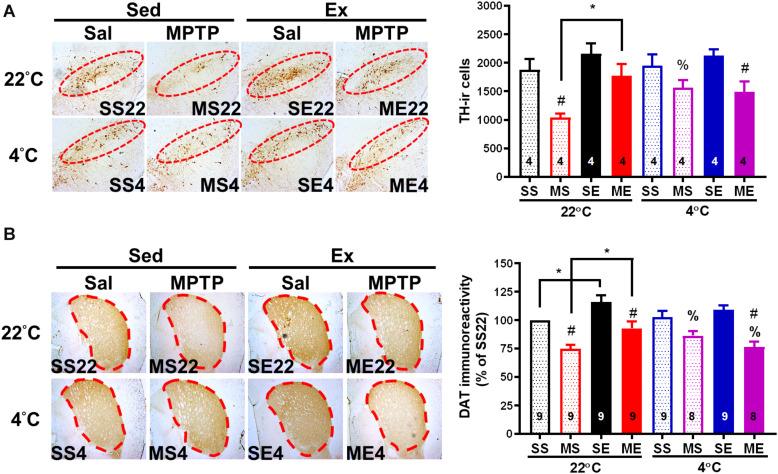
Exercise-induced neuroprotection is attenuated by cold exposure after exercise. The mice were sacrificed 24 h after the cessation of 4 weeks of exercise training. Coronal brain sections in the area of the midbrain **(A)** and striatum **(B)** obtained from the eight groups as described in previous figures were immunostained with anti-tyrosine hydroxylase (TH, dopaminergic neuron marker) antibody and anti-dopamine transporter (DAT) antibody, respectively. The TH-immunoreactive (TH-ir, brown color) cells indicating dopaminergic neurons in the SNpc (the red circles in the left panel) were counted and are shown in the right panel of **(A)**. The optical density of DAT immunoreactivity in the areas of the striatum (dotted lines in the left panel) was measured using an image tool, as shown in the right panel of **(B)**. The values are shown as means ± SEM, and the number in each column is the sample size (*n*). The treatments of SS22, MS22, SE22, ME22, SS4, MS4, SE4, and ME4 are as described in previous figures. A comparison of the two groups indicated significance (*p < 0.05) and ^#^*p* < 0.05 indicated significance as compared with the corresponding saline-treated group using a two-way ANOVA and a *post hoc* Tukey’s test. A comparison with the corresponding 22°C exposure group indicated significance after the unpaired *t*-test (^%^*p* < 0.05).

We further examined whether an improvement in motor function through exercise or cold exposure is correlated with neuroprotection against MPTP-induced toxicity. We detected the number of dopaminergic neurons by immunostaining of TH in the SNpc and DAT in the striatum of the dopaminergic nerve terminal. Four weeks after the MPTP treatment, nigrostriatal dopaminergic neurodegeneration was found, including a significantly reduced number of dopaminergic neurons (TH^ir^ cells) in SNpc [Figure 6A, MS22 vs. SS22, *F*(1,12) = 12.52, *p* = 0.0041] and DAT immunoreactivity in the striatum [Figure 6B, MS22 vs. SS22, *F*(1,33) = 30.00, *p* < 0.0001]. The MPTP-induced loss of TH^ir^ neurons in SNpc and DAT immunoreactivity in the striatum was significantly reduced in the mice co-treated with 4 weeks of exercise [ME22 vs. MS22, *F*(1,12) = 8.457, *p* = 0.0131 in Figure 6A and *F*(1,33) = 15.03 in Figure 6B, *p* = 0.0005] or daily 4°C exposure without running (Figures 6A,B, SS4 vs. SS22, *p* < 0.05, based on the unpaired *t-*test). However, in the mice treated with post-exercise 4°C exposure, the exercise-induced attenuation of MPTP-induced striatal DAT downregulation was absent (Figure 6B, ME4 vs. ME22, *p* < 0.05, based on the unpaired *t-*test), but the MPTP-induced loss of TH^ir^ neurons still existed (Figure 6A, ME4 vs. ME22). These results are in line with the motor functions and demonstrate that exercise training or daily cold adaptation has promising effects in terms of preventing MPTP-induced nigrostriatal DA neurodegeneration, while cold exposure after running attenuates exercise-induced neuroprotective effects.

## Discussion

Our results demonstrated that in addition to the induction of a transient, pronounced decrease in brain and body temperatures, mild brain hypothermia was found to last for 4 weeks in the MPTP-induced nigrostriatal dopaminergic neurodegeneration group. Preventing brain hypothermia by daily exercise training or 4°C exposure was associated with the prevention of MPTP-induced UCP4 downregulation, motor impairment, and neurotoxicity. However, cold exposure after exercise disrupted the thermoregulatory responses, UCP4 upregulation, and neuroprotection of exercise. The findings elucidated for the first time the importance of thermoregulation and striatal UCP4 upregulation in exercise-mediated neuroprotection.

In this study, we found that an MPTP injection induced a transient decrease in body core temperature that was in line with previous results of a 10% loss in subcutaneous temperature after a single injection of MPTP (Jiao et al., 2015). MPTP-induced body hypothermia has been shown to be mediated by its toxic MPP^+^ metabolite. This could not be attributed to the central effect of MPTP because intraperitoneal injection of MPP^+^, which cannot penetrate the blood–brain barrier to the brain, has also been shown to cause body hypothermia (Freyaldenhoven et al., 1995). In addition, our results demonstrated for the first time that brain temperature drops dramatically after a peripheral MPTP injection (Figure 1) and that mild brain, but not body, hypothermia was observed to last for 4 weeks after MPTP injections (Figure 2). Previous reports have shown that brain temperature becomes continuously lower in individuals with mitochondrial diseases (the failure of the respiratory chain to produce ATP similar to the inhibitory effect of MPP^+^ on complex I activity) (Rango et al., 2014), while increasing brain metabolism through the use of psychoactive drugs and intracerebroventricular administration of dopamine was found to significantly elevate brain and body temperatures (Balthazar et al., 2009; Kiyatkin et al., 2014; Zheng and Hasegawa, 2016). These findings indicate that after the penetration into the brain, MPTP is catalyzed to MPP^+^ and then induces brain hypothermia via the inhibition of mitochondrial ATP production and decreased levels of dopamine in the brain. In addition, our results indicated that preventing persistent brain hypothermia through exercise (Figure 2) or cold exposure (Figure 3) is associated with the improvement of MPTP-induced nigrostriatal dopaminergic neurodegeneration (loss of TH^ir^ neurons in the SN and reduction of DAT immunoreactivity in the striatum, Figure 6) and movement disorders (short latency to fall in rotarod performance, Figure 5). Both exercise training and cold acclimation have been demonstrated to be able to promote mitochondrial biogenesis (Marques-Aleixo et al., 2015; Pamenter et al., 2018; Salamon et al., 2019). Accordingly, lasting brain hypothermia may be the result of mitochondrial dysfunction and may contribute to MPTP-induced nigrostriatal dopaminergic neurodegeneration. On the other hand, recent reports demonstrated that TH expression is increased in preoptic area and posterior hypothalamus but unchanged in tuberoinfundibular dopaminergic pathway after MPTP treatment and that there is a positive correlation between body temperature and hypothalamic dopamine levels (Benskey et al., 2012; Zheng et al., 2018; Roostalu et al., 2019). These results suggest that the effect of MPTP on hypothalamus dopaminergic neurons may also involve in the induction of brain hypothermia. However, the low brain temperature found in this MPTP-induced mouse model of PD was distinct from a high temperature in several areas of the brains of individuals with early stage PD (Rango et al., 2016). These conflicting results may be due to the different detection system used for brain temperature and distinct progression of nigrostriatal dopaminergic neurodegeneration in human PD patients as compared with MPTP-induced Parkinsonian mice.

During treadmill running, both the body and brain temperatures of the mice were significantly elevated, and the rising amplitude of the brain temperature was only attenuated in the mice with MPTP pretreatment (Figure 2). Because brain temperature can be affected by dopamine levels and locomotor activity (Kiyatkin, 2010; Zheng et al., 2018), the decrease in exercise-induced brain hyperthermia may have been caused by the loss of dopaminergic neurons and a decrease in locomotor activity after the MPTP treatment. However, in our study, the mice were well trained and performed an equal amount of treadmill exercise daily, which was reflected in comparable body hyperthermia, and the results of open-field tests showed no differences in total distance traveled between the saline- and MPTP-treated mice with or without running (data not shown, performed before the rotarod tests). In addition, controversial results have been reported suggesting that the blockage of dopamine receptors decreases locomotion while significantly increasing brain temperature (Brown et al., 2007). Accordingly, other factors induced by exercise may contribute to brain hyperthermia, such as the increased brain metabolism related to the enhancement of mitochondrial bioenergetics and local upregulation of UCP2. For example, previous results have shown that treadmill exercise or wheel running upregulates UCP2 expression in brains through PGC-1α-regulated mitochondrial biogenesis, and local overexpression of UCP2 has been shown to elevate hypothalamic temperature (Conti et al., 2006; Dietrich et al., 2008). UCP2 upregulation was also found to be important in terms of providing the benefits of exercise in neurodegenerative diseases via a comparable effect of UCP4 and UCP5 in the reduction of oxidative stress (Chu et al., 2009; Kwok et al., 2010; de Oliveira Bristot et al., 2019). However, UCP2 knockdown in the hippocampus was not found to alter the benefits of exercise in terms of cognitive function (Wang et al., 2014), and the effects of exercise on neuronal UCP4 and UCP5 expression remain unknown. Our results showed that treadmill exercise significantly increased UCP4 protein expression and temperature in the striatum and also prevented MPTP-induced UCP4 downregulation and a low striatal temperature. Consequently, our results suggest the involvement of UCP4 upregulation in exercise-induced brain hyperthermia and neuroprotective effects. On the other hand, the possibility needs to be considered that exercise-induced UCP4 upregulation may act through its antioxidant capacity to prevent MPTP toxicity, regardless of the effects of temperature changes.

After exercise, the resting (circadian) body temperatures in the MPTP-pretreated mice were lower than those in saline-pretreated mice with post-exercise exposure to a room temperature of 22°C (Figure 4). Previous reports have demonstrated that aerobic exercise induces post-exercise hypotension due to sustained vasodilation in exercised muscle tissue (Halliwill et al., 2013), which in turns offsets exercise-induced hyperthermia (Kenny and McGinn, 2017). In the current study, we found that the low resting body temperature disappeared when post-exercise vasodilation was disrupted by inducing vasoconstriction under 4°C conditions immediately after exercise (post-exercise cold exposure). The increased vasotone due to daily 4°C exposure was consistent with the results showing that exercise-induced body hyperthermia was significantly enhanced in the mice with daily post-exercise cold exposure (Figures 2, 3). These results revealed that MPTP pretreatment may have promoted post-exercise vasodilation that led to mild body hypothermia (<0.5°C changes) while resting after exercise. The effects of MPTP on vasotone were also supported by results indicating that PD patients exhibit attenuation of resistant exercise-induced increased blood pressure (Miyasato et al., 2018) and MPTP treatment-induced loss of dopaminergic neurons in the central vasomotor areas, as well as impaired autonomic cardiovascular function (Liu et al., 2020). Besides the disruption of low resting body temperature, post-exercise 4°C exposure also blocked the decreased amplitude of exercise-induced brain hyperthermia in the MPTP-treated mice, and daily 4°C exposure alone prevented MPTP-induced brain hypothermia during the time the mice remained on the stationary treadmill (Figure 3). Considering that cutaneous vasoconstriction has been reported to be a critical factor in mediating drug-induced brain hyperthermia (Kiyatkin et al., 2014), cold exposure may induce peripheral vasoconstriction to prevent MPTP-induced brain hypothermia, decreased amplitude of brain hyperthermia during exercise, and a low resting body temperature after exercise. Once the abovementioned thermal responses are disrupted by post-exercise cold exposure, the neuroprotective effects of exercise are absent. Accordingly, our results elucidate the importance of exercise-induced thermoregulation in protecting nigrostriatal dopaminergic neurons from MPTP intoxication. In addition, we found that post-exercise 4°C exposure also disrupted the preventive effects of exercise or cold exposure on MPTP-induced UCP4 downregulation. Knockdown of UCP4 expression increases oxidative insults in primary hippocampal neurons, and UCP4 downregulation has been found in AD and PD brain tissue (Liu et al., 2006; Thangavel et al., 2017; Xu et al., 2018). Brain UCP4 mRNA expression was shown to be upregulated after acute exposure to temperatures of 4°C (Yu et al., 2000), and UCP4 overexpression has been shown to prevent ATP deficiency and oxidative insult in PD cell models (Chu et al., 2009; Wu et al., 2014; Xu et al., 2018). Mitochondrial biogenesis can be elevated by exercise training or cold adaptation (Marques-Aleixo et al., 2015; Pamenter et al., 2018; Salamon et al., 2019). Accordingly, it is reasonable to infer that the prevention of UCP4 downregulation related to mitochondrial dysfunction contributes to exercise training- or cold adaptation-induced neuroprotection against MPTP toxicity. In addition, the underlying mechanisms involved in the combination of exercise training and cold exposure in terms of blocking the respective prevention of MPTP-induced UCP4 downregulation are still unclear and worth further investigation.

## Conclusion

Our results demonstrate several lines of new information to support the involvement of post-exercise thermoregulation and UCP4 upregulation in the neuroprotective effects of exercise against MPTP toxicity. We also found that brain, but not body, temperature is low in MPTP-induced Parkinsonian mice. Daily cold adaptation has a comparable protective action to that of exercise training, while post-exercise cold exposure abrogates the benefits of exercise. Accordingly, moderate treadmill exercise or daily cold adaptation is recommended to alleviate disease progression, but post-exercise cold exposure is not suggested for PD patients. Also, because the brain temperature changes are distinct, where they are lower in MPTP-induced mouse PD models and higher in individuals with PD (Sumida et al., 2015; Rango et al., 2016), studies of other PD animal models and patients with different stages of PD are needed to further unravel the role of treadmill exercise-induced thermal changes in protecting nigrostriatal dopaminergic neurons from degeneration.

## Data Availability Statement

All datasets generated for this study are included in the article/Supplementary Material.

## Ethics Statement

The animal study was reviewed and approved by National Cheng Kung University Institutional Animal Care and Use Committee (IACUC #105094).

## Author Contributions

All authors had full access to all the data in the study and took responsibility for the integrity of the data and the accuracy of data analysis. Y-CJ and S-HC wrote the first draft of the manuscript. Y-JT and J-IC edited the manuscript. Y-JT, Y-CJ, and J-IC designed the research. Y-CJ, S-HC, Y-CL, and C-HC performed the research and analyzed the data. J-IC acquired the funding.

## Conflict of Interest

The authors declare that the research was conducted in the absence of any commercial or financial relationships that could be construed as a potential conflict of interest.
