# Supplementary material for: Cold Exposure After Exercise Impedes the Neuroprotective Effects of Exercise on Thermoregulation and UCP4 Expression in an MPTP-Induced Parkinsonian Mouse Model
Source: Front Neurosci. 2020 Sep 15;14:573509. doi: 10.3389/fnins.2020.573509 (PMC7522410; doi:10.3389/fnins.2020.573509)
Supplement: Supplementary file 1 [file Data_Sheet_1.PDF]

Supplementary Table 1. The body and brain temperature baseline in each group.

	Body temperature				Brain temperature			
	SS	MS	SE	ME	SS	MS	SE	ME
22°C	35.42±0.02	35.55±0.06	35.57±0.34	35.45±0.11	36.28±0.11	36.26±0.17	35.57±0.34	35.45±0.11
4°C	35.30±0.11	35.69±0.09	35.81±0.08	35.71±0.20	36.05±0.16	36.39±0.03	36.37±0.03	36.11±0.54

The average of daily body and brain temperatures in the period of 30 min before the mice moved to the stationary or running treadmill 5 days per week from W3 to W5 were calculated and treated as the baseline of temperature. SS represents saline-treated sedentary mice; MS represents MPTP-treated sedentary mice; SE represents saline-treated exercise mice; ME represents MPTP-treated exercise mice. After running or not running, mice were stayed at 22°C or 4°C environment for 2 hours. Value are mean ± SEM, n=3-4.

Supplementary Fig. 1

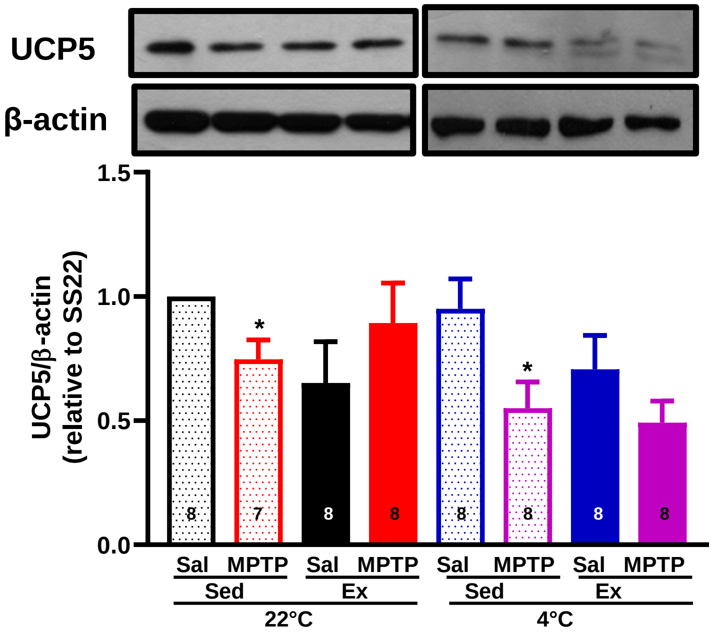

Supplementary Fig. 1. The UCP5 protein expression in the striatum after the treatments. The striatum was collected for the determination of UCP5 protein expression by western blot 24 hours after the cessation of the fourth week of treadmill exercise. The representative western bands of UCP5 and β-actin are shown in the upper panel. The optimal band intensity was determined by densitometry and the quantified data are shown in the lower panel. The values are mean ± SEM and the number indicated in each column is the sample size (n). *$p < 0.05$, as compared with the corresponding saline treated group (MS22 vs. SS22 and MS4 vs. SS4) using an up-pared *t* test. ME4 vs. ME22, $p = 0.071$, using an up-pared *t* test.
